# Copper(II)-salt-promoted oxidative ring-opening reactions of bicyclic cyclopropanol derivatives via radical pathways

**DOI:** 10.3762/bjoc.9.156

**Published:** 2013-07-11

**Authors:** Eietsu Hasegawa, Minami Tateyama, Ryosuke Nagumo, Eiji Tayama, Hajime Iwamoto

**Affiliations:** 1 Department of Chemistry, Faculty of Science, Niigata University, Ikarashi-2 8050, Niigata 950-2181, Japan

**Keywords:** copper(II) salt, cyclopropanol, electron transfer, free radical, radical ion probe

## Abstract

Copper(II)-salt-promoted oxidative ring-opening reactions of bicyclic cyclopropanol derivatives were investigated. The regioselectivities of these processes were found to be influenced by the structure of cyclopropanols as well as the counter anion of the copper(II) salts. A mechanism involving rearrangement reactions of radical intermediates and their competitive trapping by copper ions is proposed.

## Introduction

**Scheme 1:**
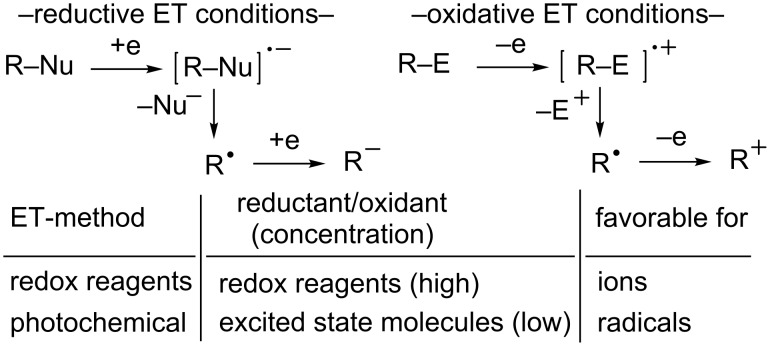
Comparison of fragmentation reaction pathways of organic radical ions generated under the redox-reagent-promoted ET and PET conditions.

**Scheme 2:**
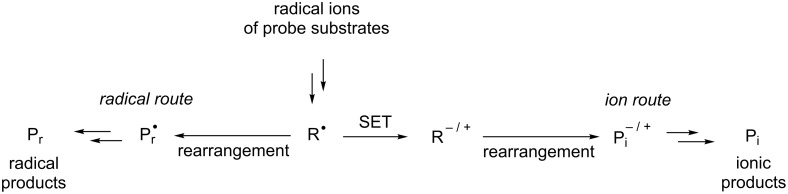
Using rearrangements of radicals and ions to distinguish mechanistic pathways for ET-reactions.

Radical ions are key intermediates in electron-transfer (ET) reactions of organic molecules [1–5] and they often undergo fragmentations to yield free radicals and ions [6–10]. The ensuing reaction pathways followed by the resulting radicals are governed not only by their intrinsic nature but also by the nature of co-existing redox reagents. In principle, radical intermediates in ET-promoted reactions have a tendency to participate in further ET processes to generate ionic species when stoichiometric amounts of redox reagents are used (Scheme 1) [1–10]. In contrast, radical intermediates formed by a photoinduced ET (PET) are less likely to undergo these secondary reactions, because steady-state concentrations of PET-generated redox reagents are low [11–19]. When radical intermediates and ions derived from their precursor radical ions undergo different rearrangement reactions, it is often possible to distinguish respective reaction pathways of radicals and ions by examining the product distributions of the reactions of substrates that contain appropriate probe moieties (Scheme 2).

**Figure 1:**
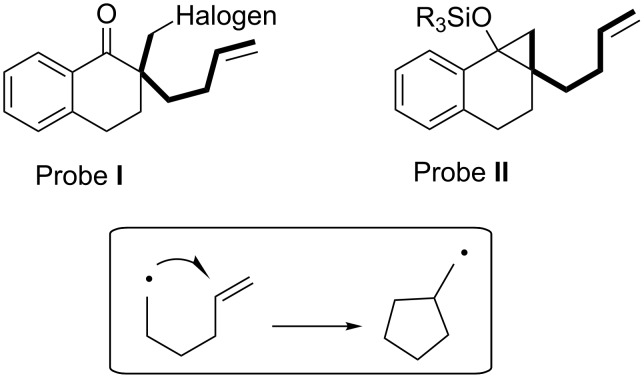
Radical anion and cation probe substances **I** and **II**, possessing 5-hexenyl structures.

**Scheme 3:**
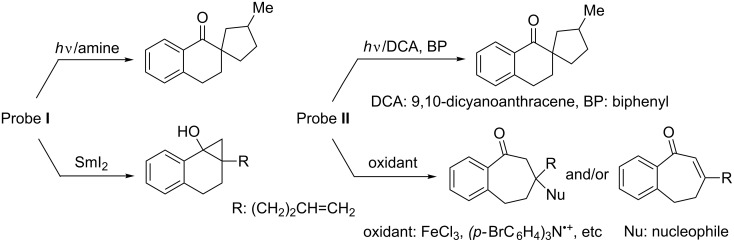
Reductive ET reactions of the probe **I** (left) and oxidative ET reactions of probe **II** (right).

In past studies, we developed unique families of substances (exemplified by probes **I** and **II** in Figure 1) that act as radical ion probes [20] and found that radical intermediates in their reaction pathways undergo efficient 5-*exo* hexenyl radical cyclization reactions [21], (Figure 1) [22–30]. For example, PET reactions of probe **I** with amines were observed to produce a spirocyclic ketone product while its reduction reaction induced by samarium diiodide (SmI_2_) gives rise to a cyclopropanol (left in Scheme 3) [22,24,27]. On the other hand, the same spirocyclic ketone is obtained in the 9,10-dicyanoanthracene (DCA) and biphenyl (BP) sensitized PET reaction of probe **II**, while reactions of this substrate with certain oxidants afford ring-expanded ketone and enone products (right in Scheme 3) [23,25–26,28–30].

Careful examination of the reaction of probe **II** with FeCl_3_ revealed that a small quantity of the spirocyclic ketone was also formed [23,28]. This observation prompted us to explore the possibility that the free radical rearrangement route becomes more predominant when oxidizing reagents weaker than Fe(III) are used to promote the reaction. Based on a consideration of the redox potentials of Fe and Cu ions (*E*º in H_2_O, V versus NHE), +0.77 for Fe(III)/Fe(II), +0.17 for copper(II)/copper(I) [31], we chose to explore the use of copper(II) reagents in this effort. Although various ET reagents have been employed to promote reactions of cyclopropanol derivatives [32–47], the employment of copper(II) reagents to induce reactions has not been extensively studied [36,39]. In the investigation described below, we have explored copper(II)-salt-promoted oxidative ring-opening reactions of selected bicyclic cyclopropanol derivatives.

## Results and Discussion

**Scheme 4:**
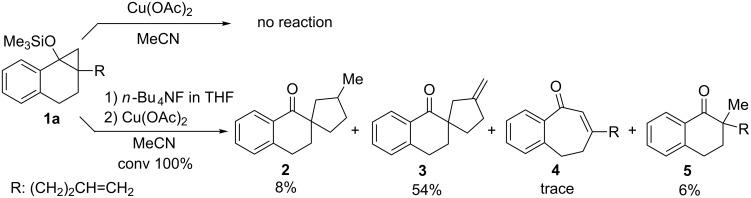
Reaction of silyl ether **1a** with Cu(OAc)_2_ in the absence or presence of *n*-Bu_4_NF.

In the initial phase of this effort, we examined the reaction of cyclopropyl silyl ether **1a** (0.40 mmol) with copper(II) acetate, Cu(OAc)_2_ , (1.1 equiv) for 1 h at room temperature (Scheme 4). Under these conditions no reaction takes place, which we attribute to the steric bulk of the silyl substituent causing interference in the reaction of the substrate with Cu(OAc)_2_. In accordance with this reasoning, we found that inclusion of *n*-Bu_4_NF (1.2 equiv) in the reaction mixture led to a reaction that completely consumes **1a** and produced the expected spirocyclic ketone **2**, albeit in low yield, and spirocyclic ketone **3** possessing an *exo*-methylene moiety as the major product. Interestingly, ketone **3** was previously observed as a product of the DCA–BP-sensitized PET reaction of **1a** in the presence of Cu(OAc)_2_ [25]. Only a trace amount of ring-expanded enone **4** along with small amounts of desilylated alcohol **1b** (ca. 8%) and ketone **5** were detected in the product mixture by using ^1^H NMR analysis. Treatment of **1a** (0.19 mmol) with *n-*Bu_4_NF (2.0 equiv) in THF for 1 h followed by hydrolysis gave a mixture of **1b** and **5** (12:88). Therefore, **5** may not result from the copper(II)-oxidation reaction.

**Scheme 5:**
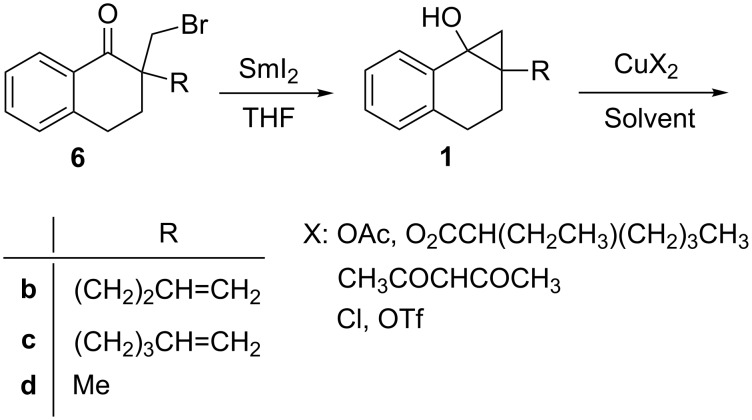
SmI_2_-promoted preparation of **1** and subsequent reaction with CuX_2_.

Based on the above observations, we anticipated that sterically less hindered cyclopropanols would more efficiently undergo copper(II)-induced oxidation reactions than the corresponding silyl ethers. To probe this prediction, cyclopropanols **1**, prepared by SmI_2_-promoted intramolecular Barbier reaction of the corresponding α-bromomethyl cycloalkanones **6** [28], were subjected to reactions promoted by various copper(II) salts, CuX_2_ (Scheme 5).

**Scheme 6:**
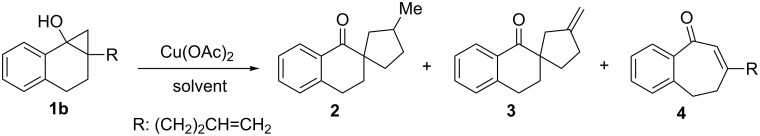
Reaction of cyclopropanol **1b** with Cu(OAc)_2_.

The results of the reaction of **1b** with Cu(OAc)_2_ (Scheme 6) are summarized in Table 1. As expected, this process produces ketone **3** as the major product along with both **2** and ring-expanded enone **4** as minor products. Moreover, the order of addition of **1b** and Cu(OAc)_2_ does not significantly affect the product distribution (compare Table 1, entry 1 to entry 2). An exploration of solvent effects revealed that MeCN is more suitable than DMF while the solubility of Cu(OAc)_2_ is higher in the latter solvent (compare Table 1, entry 1 to entry 5). In entry 5 (Table 1), ring-opened ketone **5** was obtained. In other experiments (see below), the formations of **5** (see Table 2), and other ring-opened ketones **22** (see Table 3) and **25** (see Scheme 11) are also observed. These products might be formed by deprotonation of the corresponding cyclopropanols **1**. It should be noted that THF is not an appropriate solvent for this reaction (Table 1, entry 8), a finding that is in contrast to the previous observation that ether is a better solvent than MeCN and DMF in Cu(BF_4_)_2_-promoted ring-opening reactions of cyclopropylsilyl ethers [39]. When CH_2_Cl_2_ is employed as solvent, formation of **2** becomes more efficient while the yield of **3** remains moderate (Table 1, entry 7). Although the effect of the quantity of Cu(OAc)_2_ on the reaction is not great, a decrease in the amount of Cu(OAc)_2_ causes a small increase in the yield of **2** and a decrease in the yield of **3** (compare Table 1, entry 3 to entry 1). By using more Cu(OAc)_2_, the yield of **3** is increased in DMF (compare Table 1, entry 6 to entry 5) while it is decreased in MeCN (compare Table 1, entry 4 to entry 2).

**Table 1:** Reaction of cyclopropanol **1b** with Cu(OAc)_2_.^a^

entry	Cu(OAc)_2_ (equiv)	solvent	conv of **1b**^b^ (%)	yields^c^ (%)		
				**2**	**3**	**4**

1	1.1	MeCN	91	0	70	~5^d^
2^e^	1.1	MeCN	100	0	70	~8^d^
3	0.5	MeCN	82	5	47	~2^d^
4^e^	2.2	MeCN	100	0	62	4
5^f^	1.1	DMF	60	0	35	trace
6	2.2	DMF	69	1	40	~1^d^
7^e^	1.1	CH_2_Cl_2_	85	10	38	trace
8	1.1	THF	28	trace	6	trace

^a^**1b** derived from **6b** (0.40 mmol) was added to Cu(OAc)_2_ in a solvent (4 mL). ^b^Determined by ^1^H NMR based on the yield of the isolated products (see Experimental). ^c^Isolated or determined by ^1^H NMR. ^d^Crude yields. ^e^Cu(OAc)_2_ was added to **1b** in a solvent. ^f^Ketone **5** (~5%) was obtained.

**Scheme 7:**
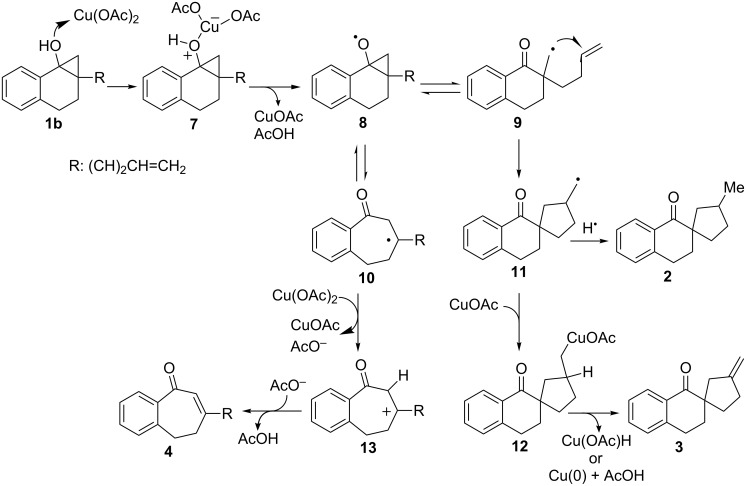
Plausible reaction pathways for the reaction of **1b** with Cu(OAc)_2_.

The observations described above suggest that the mechanism for this reaction shown in Scheme 7 is plausible. Because copper(II) is a relatively weak outer-sphere SET oxidant [1], addition of the hydroxy group of **1b** to Cu(OAc)_2_ takes place initially to produce Lewis base–acid complex **7**, followed by inner-sphere ET involving elimination of CuOAc and AcOH, which gives cyclopropoxy radical **8**. Either external or internal bond cleavage of **8** generates the respective primary alkyl radical **9** or tertiary alkyl radical **10**. An equilibrium interconverting **9** and **10** through **8** [22–30] might occur (see below). A mechanism on the fragmentation of initially formed metal–organic complexes, giving β-ketoalkyl radicals [40], cyclopropoxy radicals [25,28,48–50], or β-metalated carbonyls [39], is still controversial [35–47]. Thus, we believe the reaction follows the pathways shown in Scheme 7 although the possibility of direct formations of **9** and **10**, a concerted ET and cyclopropane ring opening, cannot be ruled out. Rapid 5-*exo* cyclization of hexenyl radical moiety in **9** produces spirocyclic primary alkyl radical **11**. Hydrogen-atom abstraction by **11** then leads to formation of spirocyclic ketone product **2**, while trapping of **11** by CuOAc followed by β-H elimination (either hydride elimination or deprotonation) [39] of the resulting organocopper intermediate **12** generates the exocyclic methylene analogue **3** as the major product [25]. Protonation of **12** might be an alternative route for the formation of **2** (not shown in Scheme 7). Reactions of alkyl radicals with copper(II) are well documented [51–52], and it has been also suggested that copper(I) efficiently reacts with alkyl radicals [39]. As described, 1.1 equiv of Cu(OAc)_2_ leads to nearly complete reaction of **1b** (see entry 1 and entry 2 in Table 1). Thus, CuOAc which is generated after initial ET between Cu(OAc)_2_ and **1b** may capture the primary alkyl radical **11**. In addition, although not predominant, oxidation of **10** by Cu(OAc)_2_ gives rise to tertiary carbocation **13** [51–52], which is then deprotonated to form enone **4**.

**Scheme 8:**
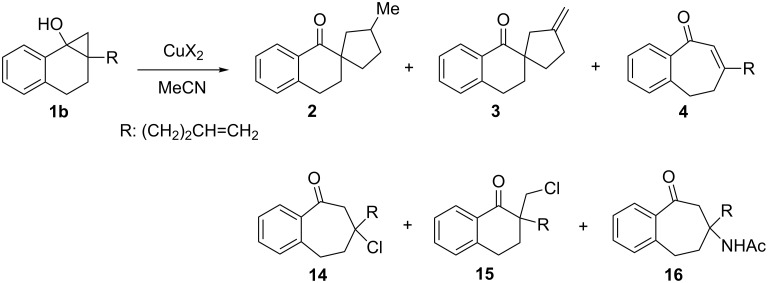
Reaction of cyclopropanol **1b** with various copper(II) salts (CuX_2_).

**Scheme 9:**
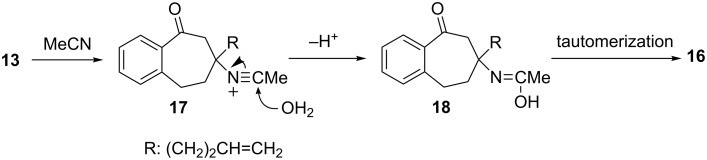
Formation of acetoamide **16** from the cation **13**.

Studies of the effect of the counter ion on copper(II)-promoted reactions of **1b** (Scheme 8) gave the results summarized in Table 2. While no reaction occurred when copper(II) acetylacetonate, Cu(acac)_2_, is used, (Table 2, entry 1), copper(II) 2-ethylhexanoate, Cu(ehex)_2_, serves as an effective oxidant in transforming **1b** to **3** in a yield that is comparable to the process promoted by Cu(OAc)_2_ (compare Table 2, entry 2 to entry 3). Noticeable amounts of **2** are also generated in this reaction. When CuCl_2_ is employed to oxidize **1b**, only ring-expanded ketones **4** and **14** are produced along with a lesser amount of chloro ketone **15**, and competitive formation of **2** and **3** does not occur (Table 2, entry 4). An increase in the amount of CuCl_2_ causes a slight increase in the conversion of **1b** and the total yield of ring-expanded products **4** and **14** (compare Table 2, entry 5 to entry 4). Interestingly, CuCl_2_ (1.1 equiv) could also promote the reaction of silyl ether **1a** to produce **4** (23%), **14** (4%) and **15** (3%) at 89% conversion of **1a**. Although the origin of **15** is uncertain, one possibility is that it is formed by halogen substitution of unconverted bromide **6b** to **1b** by SmI_2_. The formation of chloro ketone **23** (see Table 3) may be similarly explained. Finally, reaction of **1b** with Cu(OTf)_2_ leads to formation of ring-expanded products **4** and **16** and a negligible amount of **2** (Table 2, entry 6). Acetamide **16** is probably produced in this process through a Ritter reaction between cation **13** and the solvent acetonitrile (Scheme 9).

**Table 2:** Reaction of cyclopropanol **1b** with various copper(II) salts (CuX_2_).^a^

entry	X	Conv of **1b**^b^ (%)		yields^c^ (%)	
			**2**	**3**	**4**

1	acetyl acetonate (acac)	0		No reaction	
2^d^	2-ethyl hexanoate (ehex)	94	5	63	~4^e^
3^f^	OAc	91	0	70	~5^e^
4^g^	Cl	63	0	0	~25^e^(9)^h^
5^i^	Cl	71	0	0	34(6)^h^
6^j^	OTf	77	trace	0	~11^e^(34)^k^

^a^**1b** derived from **6b** (0.40 mmol) was added to CuX_2_ (1.1 equiv for entries 1–4,6; 2.2 equiv for entry 5) in MeCN (4 mL). ^b^Determined by ^1^H NMR based on the yield of the isolated products (see Experimental). ^c^Isolated or determined by ^1^H NMR. ^d^Ketone **5** (13%) was obtained. ^e^Crude yield. ^f^Same as entry 1 in Table 1. ^g^Ketone **5** (9%) and chloro ketone **15** (4%) were obtained. ^h^Number in the parenthesis is the yield of chloro adduct **14**. ^i^Ketone **5** (13%) and chloro ketone **15** (11%) were obtained. ^j^Ketone **5** (~2%) was obtained. ^k^Number in parentheses is the yield of acetoamide **16**.

Hypothetically, both the Lewis acidity and oxidizing ability of CuX_2_ should depend on the basicity of the counter ion (X^−^: conjugate base of HX). Based on the acidity order HX, TfOH > HCl > AcOH ~ 2-ethyl hexanoic acid > acetylacetone [53–54], it is possible to assign Cu(acac)_2,_ which is ineffective in promoting the reaction, as the weakest oxidant. On the other hand, CuCl_2_ and Cu(OTf)_2_ induce reactions that follow a different pathway from those promoted by copper(II) carboxylates. These observations suggest that a rapid equilibrium does indeed exist between isomeric radical intermediates **9** and **10** (Scheme 7) and that the thermodynamically less stable isomer **9** undergoes fast hexenyl-radical cyclization leading to the formation of **11** in reactions promoted by copper(II) carboxylates. On the other hand, a fast oxidation of the more stable isomer **10** by stronger oxidants such as CuCl_2_ or Cu(OTf)_2_ occurs to give the stable tertiary carbocation **13**, which is then captured by Cl^−^ or MeCN.

**Scheme 10:**
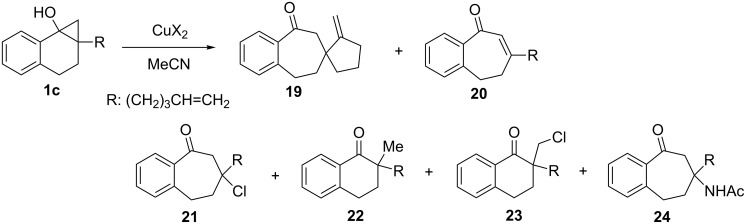
Reaction of cyclopropanol **1c** with various copper(II) salts (CuX_2_).

In order to explore the generality of the proposed counter-anion-dependent reactivity switch in the nature of copper(II)-promoted reactions of **1**, the pentenyl-substituted cyclopropanol **1c** was employed as the substrate (Scheme 10 and Table 3). A major product of the reaction of **1c** promoted by Cu(OAc)_2_ was observed to be the *exo*-methylene containing spirocyclic ketone **19** (Table 3, entry 1), which is produced in the DCA–BP sensitized PET reaction of silyl ether of **1c** in the presence of Cu(OAc)_2_ [25]. Contrary to the expectation that a base could assist the deprotonation of the complex between copper and **1c** (similar to **7** in Scheme 7), the addition of pyridine was found to decelerate the reaction (Table 3, entry 2). This observation suggests that coordination of pyridine to copper reduces the oxidizing ability of Cu(OAc)_2_. Cu(ehex)_2_ was also effective to give **19** although the yield was relatively low (Table 3, entry 3). Reaction of **1c** with CuCl_2_ was observed to form ring-expanded ketones **20** and **21**, along with small amounts of **22** and **23**. However, competitive generation of **19** does not take place (Table 3, entry 4). Finally, reaction of **1c** with Cu(OTf)_2_ leads to the formation of ring-expanded enone **20** and acetoamide **24** (Table 3, entry 5).

**Table 3:** Reaction of cyclopropanol **1c** with various copper(II) salts (CuX_2_).^a^

entry	X	additive	conv of **1c**^b^ (%)	yields^c^ (%)	
				**19**	**20**

1	OAc	–	95	55	0
2	OAc	pyridine (1.2 equiv)	~65^d^	33	0
3	ehex	–	100	33	0
4^e^	Cl	–	63	0	28(8)^f^
5	OTf	–	~93^d^	0	13(33)^g^

^a^CuX_2_ (1.1 equiv) was added to **1c** derived from **6c** (0.4 mmol) in MeCN (4 mL). ^b^Determined by ^1^H NMR based on the yield of the isolated products (see Experimental). ^c^Isolated or determined by ^1^H NMR. ^d^Based on the crude yield of **1c**. ^e^Ketone **22** (14%) and chloro ketone **23** (5%) were obtained. ^f^Number in parentheses is the yield of chloro adduct **21**. ^g^Number in parentheses is the yield of acetoamide **24**.

**Scheme 11:**
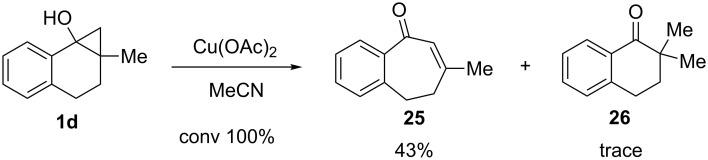
Reaction of cyclopropanol **1d** with various Cu(OAc)_2_.

As described above, observation of the occurrence of hexenyl-radical cyclization processes serves as good evidence for the involvement of radical intermediates in mechanistic pathways for reactions of **1b** and **1c**. In order to gain more information about these processes, we explored an oxidation reaction of substrate **1d**, which does not contain an alkene tether and whose reaction pathway, thus, cannot involve radical intermediates that undergo hexenyl-radical cyclization. We observed that reaction of the methyl-substituted cyclopropanol **1d** with Cu(OAc)_2_ leads to formation of the ring-expanded enone **25** as a major product along with a trace amount of ketone **26** (Scheme 11).

**Scheme 12:**
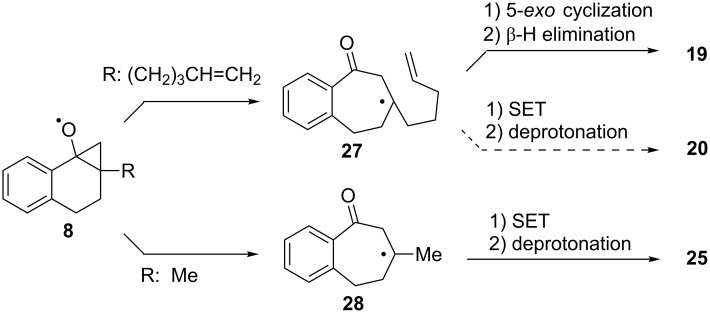
Comparison of reaction pathways of ring-expanded radical **27** and **28**.

The Cu(OAc)_2_-promoted reactions of **1c** and **1d** are compared in Scheme 12. The ring-expanded tertiary alkyl radical **27**, formed as an intermediate in the reaction of **8** (R = (CH_2_)_3_CH=CH_2_), undergoes rapid 5-*exo* hexenyl cyclization along the route for the production of spirocyclic ketone **19**. Thus, oxidation of **27** followed by deprotonation to give enone **20** is a minor contributor. If an external bond cleavage of **8** occurs, cyclization of heptenyl-radical moiety in the resulting primary alkyl radical (not shown in Scheme 12) is expected. However, the *exo*-cyclization of heptenyl radical is two orders of magnitude slower than that of the hexenyl radical [55]. In contrast, because no competitive radical-rearrangement process exists, the corresponding radical intermediate **28** formed from **8** (R = Me) undergoes sequential oxidation and deprotonation to give enone **25** as a major product.

## Conclusion

Various copper(II) salts promote ring-opening reactions of bicyclic cyclopropanol derivatives. Using substrates that possess hexenyl moieties, we observed that the nature of the counter anion of copper(II) salts has a significant impact on the product distributions. The results suggest that reaction pathways followed by radical intermediates derived from these substrates are strongly influenced by post ring-opening steps. Thus, cyclopropane bond cleavage, which is reversible, does not serve as a product-determining step if a rapid follow-up reaction like hexenyl-radical cyclization does not exist. The results show that by using a proper choice of copper(II) salts it is possible to control the reaction pathways followed by radical and ionic intermediates derived from the initially formed Lewis base–acid complexes if the radicals and ions are capable of undergoing different rearrangement reactions.

## Experimental

**General:** NMR spectra were recorded in CDCl_3_ with Me_4_Si as an internal standard at 400 MHz for ^1^H NMR and 100 MHz for ^13^C NMR. Column chromatography was performed with silica gel (Wakogel C-200). Preparative TLC was performed on 20 cm × 20 cm plates coated with silica gel (Wakogel B-5F). MeCN was distilled over P_2_O_5_ and subsequently distilled with K_2_CO_3_. CH_2_Cl_2_ was treated with H_2_SO_4_, water, 5% NaOH, water, and CaCl_2_, and then distilled with CaH_2_. THF was distilled over sodium benzophenone under N_2_. Anhydrous DMF was purchased and used without distillation. Other reagents and solvents were purchased and used without further purification. Substrates **1a** [25], **1b** [29], **1d** [29], **6b** [24], and **6d** [28] and products **2** [24], **3** [25], **4** [25], **5** [29], **19** [25], **20** [26], **25** [25], and **26** [25] are known compounds. Spectral data of **1c**, **6c**, **14**, **15**, **16**, **21**, **22**, and **23** are presented below.

**Preparation of cyclopropanols 1:** Cyclopropanol derivatives **1** were prepared from the corresponding bromo ketones **6** by using SmI_2_ following previously reported procedures [25,28]. Silyl ether **1a** was prepared by the treatment of alcohol **1b** with TMSCl and Et_3_N. The synthesized alcohols **1b**, **1c** and **1d** were directly used for the reactions owing to their instabilities during silica-gel chromatography.

**1-Hydroxy-3-(4-pentenyl)-6,7-benzobicyclo[4.1.0]heptane (1c):** White solid; mp 71.5–72.9 °C; ^1^H NMR (400 MHz, CDCl_3_) δ 7.67–7.64 (m, 1H), 7.23–7.17 (m, 1H), 7.11–7.05 (m, 1H), 7.03–6.99 (m, 1H), 5.88–5.76 (m, 1H), 5.05–4.93 (m, 2H), 2.62 (ddd, *J* = 15.2, 5.2, 1.6 Hz, 1H), 2.52 (bs, 1H), 2.38 (td, *J* = 15.2, 5.2 Hz, 1H), 2.14–2.04 (m, 2H), 1.96 (ddd, *J* = 12.8, 5.6, 2.0 Hz, 1H), 1.66–1.46 (m, 5H), 1.20 (d, *J* = 6.0 Hz, 1H), 0.81 (d, *J* = 5.6 Hz, 1H); ^13^C NMR (100 MHz, CDCl_3_) δ 140.8, 138.9, 133.0, 127.9, 126.2, 125.3, 123.8, 114.4, 58.6, 33.9, 32.1, 30.5, 26.9, 26.4, 23.3, 21.3; IR (neat) ν_max_ (cm^−1^): 3278, 3188, 3072, 2921, 1640, 1444, 1278, 1228, 1194, 990, 908, 740; LRMS–EI *m*/*z* (% relative intensity): 228 (M^+^, 6), 160 (100); HRMS–EI (*m*/*z*): [M]^+^ calcd for C_16_H_20_O, 228.1514; found, 228.1511.

**2-Bromomethyl-2-(4-pentenyl)-1-tetralone (6c):** Pale yellow oil; ^1^H NMR (400 MHz, CDCl_3_) δ 8.05–8.02 (m, 1H), 7.51–7.46 (m, 1H), 7.34–7.23 (m, 2H), 5.77–5.70 (m, 1H), 5.00–4.91 (m, 2H), 3.77 (d, *J* = 10.4 Hz, 1H), 3.64 (d, *J* = 10.4 Hz, 1H), 3.13–2.90 (m, 2H), 2.34–2.16 (m, 2H), 2.04–1.98 (m, 2H), 1.78–1.24 (m, 4H); ^13^C NMR (100 MHz, CDCl_3_) δ 198.7, 143.0, 138.0, 133.5, 131.3, 128.8, 128.1, 126.8, 115.0, 48.6, 39.3, 33.9, 32.7, 30.9, 24.8, 22.7; IR (neat) ν_max_ (cm^−1^): 2938, 1680, 1600, 1454, 1304, 1224, 991, 910, 743; HRMS–ESI (*m*/*z*): [M + H]^+^ calcd for C_16_H_19_O^79^Br, 307.0692; found, 307.0687; [M + H]^+^ calcd for C_16_H_19_O^81^Br, 309.0672; found, 306.0665.

**Reaction of cyclopropanols 1 with copper(II) salts:** A typical experiment using **1b** is described (Table 1, entry 1). To Cu(OAc)_2_ (79.9 mg, 0.44 mmol) in MeCN (4 mL) was added **1b** (85.7 mg, 0.40 mmol). In some experiments, the order of addition was reversed (see entry 2 in Table 1 and Table 3). The resulting mixture was stirred under N_2_ at room temperature for 1 h, diluted with water and extracted with Et_2_O. The extract was washed with water, saturated aqueous Na_2_S_2_O_3_, saturated aqueous NaHCO_3_, and brine, dried over anhydrous MgSO_4_, and concentrated in vacuo giving a residue that was subjected to TLC (AcOEt:*n*-hexane 20/1), and **3** (59.3 mg, 0.28 mmol, 70%) and **4** (~5 mg, ~0.02 mmol, ~5%) were obtained. Other reactions were performed in a similar manner. Because cyclopropanols **1** have a tendency to partially decompose during silica-gel chromatography, their conversion in reactions was determined by using ^1^H NMR analysis of the crude reaction mixtures. When product isolations were not performed, yields were also determined by ^1^H NMR, and crude yields are reported in some cases.

**3-(3-Butenyl)-3-chloro-1-benzosuberone (14):** Brown liquid; ^1^H NMR (400 MHz, CDCl_3_) δ 7.80 (d, *J* = 7.6 Hz, 1H), 7.41 (t, *J* = 6.6 Hz, 1H), 7.32–7.29 (m, 2H), 5.88–5.78 (m, 1H), 5.10–4.98 (m, 2H), 3.51–3.40 (m, 2H), 3.16 (dd, *J* = 12.0, 1.6 Hz, 1H), 3.03 (dd, *J* = 17.0, 8.2 Hz, 1H), 2.55 (dd, *J* = 15.4, 8.2 Hz, 1H), 2.47–2.38 (m, 1H), 2.34–2.25 (m, 1H), 2.10–1.96 (m, 3H); ^13^C NMR (100 MHz, CDCl_3_) δ 197.8, 143.9, 137.5, 137.2, 132.0, 130.3, 128.9, 126.5, 115.2, 72.8, 55.8, 43.1, 42.8, 31.0, 28.7; IR (neat) ν_max_ (cm^−1^): 2939, 1681, 1602, 1453, 1299, 1226, 915, 749; HRMS–ESI (*m*/*z*): [M + H]^+^ calcd for C_15_H_17_O^35^Cl, 249.1041; found, 249.1038; [M + H]^+^ calcd for C_15_H_17_O^37^Cl, 250.1074; found, 250.1071.

**2-(3-Butenyl)-2-chloromethyl-1-tetralone (15):** Colorless oil; ^1^H NMR (400 MHz, CDCl_3_) δ 8.03 (d, *J* = 8.4 Hz, 1H), 7.48 (t, *J* = 6.8 Hz, 1H), 7.31 (t, *J* = 7.6 Hz, 1H), 7.23 (d, *J* = 7.6 Hz, 1H), 5.78–5.68 (m, 1H), 5.01–4.91 (m, 2H), 3.87 (d, *J* = 11.6 Hz, 1H), 3.79 (d, *J* = 11.2 Hz, 1H), 3.13–3.05 (m, 1H), 2.96 (dt, *J* = 17.4, 5.0 Hz, 1H), 2.35–2.28 (m, 1H), 2.22–2.06 (m, 2H), 2.01–1.92 (m, 1H), 1.88–1.72 (m, 2H); ^13^C NMR (100 MHz, CDCl_3_) δ 198.8, 142.8, 137.6, 133.5, 131.3, 128.7, 128.0, 126.8, 115.0, 49.1, 49.0, 31.6, 29.9, 27.7, 24.7; IR (neat) ν_max_ (cm^−1^) 2940, 1680, 1601, 1453, 1225, 914, 748; HRMS–ESI (*m*/*z*): [M + H]^+^ calcd for C_15_H_17_O^35^Cl, 249.1041; found, 249.1041; [M + H]^+^ calcd for C_15_H_17_O^37^Cl, 251.1011; found, 251.1006.

**3-(Acetylamino)-3-(3-butenyl)-1-benzosuberone (16):** Yellow solid; mp 105.0–107.0 °C; ^1^H NMR (400 MHz, CDCl_3_) δ 7.77 (d, *J* = 6.4 Hz, 1H), 7.41 (t, *J* = 6.8 Hz, 1H), 7.30 (t, *J* = 6.4 Hz, 1H), 7.25 (d, *J* = 8.0 Hz, 1H), 5.87–5.77 (m, 1H), 5.56 (bs, 1H), 5.06–4.95 (m, 2H), 3.12–2.97 (m, 4H), 2.46–2.40 (m, 1H), 2.27–2.20 (m, 1H), 2.10–2.03 (m, 3H), 1.99–1.95 (m, 4H); ^13^C NMR (100 MHz, CDCl_3_) δ 201.3, 169.9, 144.2, 138.2, 138.1, 132.1, 130.3, 128.6, 126.6, 115.0, 57.5, 50.9, 39.3, 36.1, 31.2, 28.3, 24.2; IR (neat) ν_max_ (cm^−1^) 3308, 3209, 2246, 1665, 1599, 1548, 1450, 1298, 1232, 912, 732; HRMS–ESI (*m*/*z*): [M + Na]^+^ calcd for C_17_H_21_NO_2_, 271.1567; found, 294.1463.

**3-Chloro-3-(4-pentenyl)-1-benzosuberone (21):** Brown oil; ^1^H NMR (400 MHz, CDCl_3_) δ 7.81 (d, *J* = 7.6 Hz, 1H), 7.42 (t, *J* = 7.6 Hz, 1H), 7.32–7.26 (m, 2H) , 5.87–5.76 (m, 1H), 5.07–4.96 (m, 2H), 3.51–3.41 (m, 2H), 3.16 (dd, *J* = 12.0, 1.6 Hz, 1H), 3.03 (dd, *J* = 16.0, 8.0 Hz, 1H), 2.55 (dd, *J* = 15.4, 8.4 Hz, 1H), 2.13–2.08 (m, 2H), 1.98–1.62 (m, 5H); ^13^C NMR (100 MHz, CDCl_3_) δ 198.0, 144.0, 138.0, 137.6, 132.0, 130.3, 128.9, 126.5, 115.0, 73.3, 55.8, 43.2, 43.1, 33.4, 31.1, 23.6; IR (neat) ν_max_ (cm^−1^) 2943, 1680, 1600, 1449, 1297, 913, 751; HRMS–ESI (*m*/*z*): [M + H]^+^ calcd for C_16_H_19_O^35^Cl, 263.1197; found, 263.1191; [M + H]^+^ calcd for C_16_H_19_O^37^Cl, 265.1168; found, 265.1168.

**2-Methyl-2-(4-pentenyl)-1-tetralone (22):** Pale yellow oil; ^1^H NMR (400 MHz, CDCl_3_) δ 8.03 (d, *J* = 8.0 Hz, 1H), 7.45 (t, *J* = 7.6 Hz, 1H), 7.30 (t, *J* = 8.0 Hz, 1H), 7.21 (d, *J* = 8.0 Hz, 1H), 5.82–5.72 (m, 1H), 3.00–2.94 (m, 2H), 2.11–2.00 (m, 1H), 1.96–1.89 (m, 3H), 1.71–1.62 (m, 1H), 1.56–1.48 (m, 1H), 1.43–1.34 (m, 2H), 1.18 (s, 3H); ^13^C NMR (100 MHz, CDCl_3_) δ 202.6, 143.3, 138.6, 132.9, 131.7, 128.6, 128.0, 126.6, 114.6, 44.6, 35.9, 34.2, 33.6, 25.4, 23.3, 22.2; IR (neat) ν_max_ (cm^−1^): 2933, 2859, 1682, 1601, 1454, 1222, 909, 741; HRMS–ESI (*m*/*z*): [M + H]^+^ calcd for C_16_H_20_O, 229.1587; found, 229.1593.

**2-Chloromethyl-2-(4-pentenyl)-1-tetralone (23):** Pale yellow oil; ^1^H NMR (400 MHz, CDCl_3_) δ 8.03 (d, *J* = 8.0 Hz, 1H), 7.48 (t, *J* = 7.6 Hz, 1H), 7.31 (t, *J* = 8.0 Hz, 1H), 7.23 (d, *J* = 7.6 Hz, 1H), 5.78–5.68 (m, 1H), 5.00–4.90 (m, 2H), 3.82 (d, *J* = 11.2 Hz, 1H), 3.77 (d, *J* = 11.2 Hz, 1H), 3.12–3.04 (m, 1H), 2.95 (dt, *J* = 18.0, 4.8 Hz, 1H), 2.34–2.28 (m, 1H), 2.22–2.16 (m, 2H), 2.03–1.97 (m, 2H), 1.78–1.72 (m, 2H), 1.45–1.30 (m, 2H); ^13^C NMR (100 MHz, CDCl_3_) δ 199.1, 142.9, 138.0, 133.5, 131.4, 128.7, 128.0, 126.8, 115.0, 49.2, 49.1, 33.9, 31.9, 29.9, 24.7, 22.7; IR (neat) ν_max_ (cm^−1^): 2939, 1680, 1600, 1454, 1222, 911, 746; HRMS–ESI (*m*/*z*): [M + H]^+^ calcd for C_16_H_19_O^35^Cl, 263.1195; found, 263.1197; [M + H]^+^ calcd for C_16_H_19_O^37^Cl, 265.1168; found, 265.1168.

**3-(*N*-Acetylamino)-3-(4-pentenyl)-1-benzosuberone (24):** Viscous yellow oil; ^1^H NMR (400 MHz, CDCl_3_) δ 7.77 (d, *J* = 7.6 Hz, 1H), 7.40 (t, *J* = 7.6 Hz, 1H), 7.28 (t, *J* = 7.6 Hz, 1H), 7.24 (d, *J* = 7.2 Hz, 1H), 5.84–5.74 (m, 1H), 5.55 (bs, 1H), 5.04–4.94 (m, 2H), 3.11–2.96 (m, 4H), 2.42–2.36 (m, 1H), 2.11–2.04 (m, 4H), 1.93 (s, 3H), 1.91–1.83 (m, 1H) , 1.43–1.35 (m, 2H) ppm; ^13^C NMR (100 MHz, CDCl_3_) δ 201.3, 169.8, 144.2, 138.4, 138.2, 132.0, 130.3, 128.5, 126.5, 114.8, 57.6, 50.9, 39.2, 36.6, 33.7, 31.2, 24.2, 23.1 ppm; IR (neat) ν_max_ (cm^−1^): 3301, 3204, 2246, 1660, 1599, 1547, 1449, 1298, 1229, 912, 731; HRMS–ESI (*m*/*z*): [M + Na]^+^ calcd for C_18_H_23_NO_2_, 308.1621; found, 308.1622.
